# Primary carnitine deficiency – diagnosis after heart transplantation: better late than never!

**DOI:** 10.1186/s13023-020-01371-2

**Published:** 2020-04-10

**Authors:** Sarah C. Grünert, Sara Tucci, Anke Schumann, Meike Schwendt, Gwendolyn Gramer, Georg F. Hoffmann, Michelle Erbel, Brigitte Stiller, Ute Spiekerkoetter

**Affiliations:** 1 Department of General Paediatrics, Adolescent Medicine and Neonatology, Medical Centre-University of Freiburg, Faculty of Medicine, Mathildenstraße 1, 79106 Freiburg, Germany; 2 Department of Congenital Heart Disease and Paediatric Cardiology, University Heart Centre Freiburg - Bad Krozingen, Medical Center - University of Freiburg, Faculty of Medicine, Freiburg, Germany; 3 University Hospital Heidelberg, Centre for Paediatric and Adolescent Medicine, Division of Neuropediatrics and Metabolic Medicine, Im Neuenheimer Feld 430, 69120 Heidelberg, Germany; 4 Institute of Pathology, Medical Centre-University of Freiburg, Faculty of Medicine, Freiburg, Germany

**Keywords:** Primary carnitine deficiency, OCTN2, SLC22A5, Cardiomyopathy, Heart transplantation

## Abstract

**Background:**

Primary carnitine deficiency due to mutations in the *SLC22A5* gene is a rare but well-treatable metabolic disorder that puts patients at risk for metabolic decompensations, skeletal and cardiac myopathy and sudden cardiac death.

**Results:**

We report on a 7-year-old boy diagnosed with primary carnitine deficiency 2 years after successful heart transplantation thanks his younger sister’s having been identified via expanded newborn screening during a pilot study evaluating an extension of the German newborn screening panel.

**Conclusion:**

As L-carnitine supplementation can prevent and mostly reverse clinical symptoms of primary carnitine deficiency, all patients with cardiomyopathy should be investigated for primary carnitine deficiency even if newborn screening results were unremarkable.

## Background

Primary carnitine deficiency (PCD) is a rare autosomal recessive disorder of the carnitine cycle that results in impaired long-chain fatty acid oxidation. It is caused by mutations in the *SLC22A5* gene encoding for the organic cation transporter novel 2 (OCTN2) [1]. This high affinity carnitine transporter is expressed in many tissues including the heart, kidney, placenta, and brain [2]. Defective carnitine transport results in urinary carnitine wasting, low serum carnitine levels, and decreased intracellular carnitine accumulation [1, 3]. Patients with primary carnitine deficiency lose up to 95% of the filtered carnitine in urine [1, 4]. Since carnitine is essential for the transfer of long-chain fatty acids from the cytoplasm to mitochondrial matrix for subsequent β-oxidation, carnitine deficiency results in an impaired ability to use fat as an energy source during periods of metabolic stress [1].

PCD is a target disease of newborn screening programmes in several countries. In Germany, PCD is not included in our nation-wide newborn screening panel [5]. However, as the respective marker free carnitine (C0) is required when screening for other conditions in the German newborn screening panel, markedly decreased C0 levels are reported by newborn screening laboratories as incidental findings. Pilot studies are now evaluating an extension of the newborn screening panel to include PCD also in individual screening laboratories in Germany [6, 7]. However, the New Zealand newborn screening programme just recently decided to discontinue screening for PCD; their reasons included poor sensitivity, a high false-positive rate, and numerous asymptomatic adults with PCD [8].

The diagnosis of PCD can be confirmed via mutation analysis of the *SLC22A5* gene or carnitine uptake studies in patients’ fibroblasts [1]. Under the conditions involving low plasma carnitine levels, carnitine excretion in urine can be normal and may not constitute a diagnostic marker requiring a carnitine challenge for diagnostic purposes. Since carnitine can also be low in the plasma of vegetarians or vegans, low carnitine concentrations are found in newborns from vegetarian mothers, thus requiring further diagnostic studies in them as well.

The clinical phenotype is broad, and symptoms can vary widely with respect to age of onset, organ involvement, and severity of symptoms [4]. Patients with PCD can present with hypoketotic hypoglycaemia and hepatic encephalopathy during infancy and early childhood, or later in life with skeletal and cardiac myopathy or sudden death from cardiac arrhythmia, usually triggered by fasting or other catabolic conditions [1]. Many individuals with PCD, however, remain asymptomatic throughout their lives. Several asymptomatic mothers have been diagnosed following the birth of an unaffected child presenting abnormally low carnitine levels in newborn screening.

Patients with PCD respond well to oral L-carnitine supplementation if started before irreversible organ damage has occurred [3]. However, even under treatment, carnitine plasma levels often remain below the normal range. The dose must be adapted individually by serial carnitine measurements in plasma, but high doses of 100–400 mg/kg/day are usually required [1, 4]. In OCTN2-deficient patients, carnitine is absorbed by the gut and enters body cells through the amino acid transporter B^0,+^ (ATB^0,+^) that has less affinity towards carnitine than OCTN2, but which exhibits much higher capacity than OCTN2 [1]. Metabolic decompensations are reduced or prevented and skeletal and cardiac muscle function improve with L-carnitine supplementation [4]. The long-term prognosis is favourable as long as children and adults remain on carnitine treatment [1, 3]. However, compliance with long-term carnitine supplementation may be inadequate, and potential side-effects entailing elevated levels of trimethylamine-N-oxide (TMAO) resulting from high-dose carnitine supplementation have recently been reported [8].

We report on a 7-year-old boy who underwent cardiac transplantation due to severe cardiomyopathy at age 5 years; he was diagnosed with PCD via family screening 2 years after transplantation. His diagnosis was triggered by the identification of his younger sister through expanded newborn screening during a pilot study evaluating an extension of the German newborn screening panel.

## Results

### Case report

**Fig. 1:**
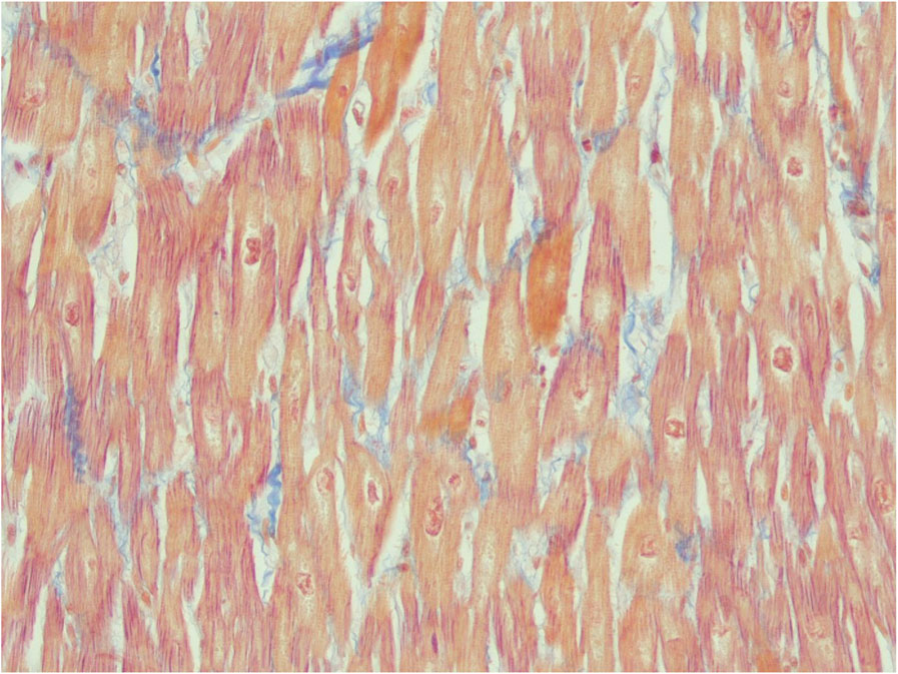
Myocardial histology of patient 1: Acid Fuchsin Orange G staining of myocardium [× 200] showing hypertrophy with thickened muscular fibres

Patient 1 is the third child of consanguineous Macedonian parents. At the age of 21 months, cardiomegaly was revealed in a chest X-ray taken due to a pulmonary infection. Regular follow-up echocardiographs showed only slight reduction in left ventricular function (LV-EF 50–55%). At age 4 years (body weight 22 kg), he was admitted to our clinic in cardiogenic shock due to dilative cardiomyopathy and severe left myocardial failure. One week later, after ICU therapy led to moderate recovery, the child went into cardiac arrest during myocardial biopsy in the cath-lab. After conventional resuscitation had failed, extracorporeal cardiorespiratory resuscitation (eCPR) with open chest cannulation was successful. Two days later, with pulmonary recovery, extracorporeal life support was replaced by a left ventricular assist device, Berlin Heart Excor, 25 ml (BerlinHeart, Germany) for long-term support and recovery. Awake and with no neurological sequelae, he was listed for heart transplantation (HTx). After almost 10 months on the BerlinHeart, HTx was uneventful and six weeks later he was discharged home. Routine follow-up investigations consistently revealed good graft function. His initial biopsy’s histology was characterised by massive endocardial fibrosis, and the biopsy of the explanted heart revealed biventricular hypertrophy with thickened muscular fibres (Fig. 1).

His younger sister (patient 2) was born two years after his HTx. In addition to newborn screening in line with the German newborn screening panel, this child participated in the pilot study “Newborn Screening 2020” at the newborn screening centre in Heidelberg, Germany. Since 2016, this study has been evaluating the possible extension of the German newborn screening panel by 26 additional conditions including PCD [7]. Newborn screening via tandem mass spectrometry in this study’s protocol revealed severely reduced levels of free carnitine. Further biochemical work-up led to the diagnosis of primary carnitine deficiency (Table 1). Maternal carnitine deficiency was excluded. Mutation analysis of the *SLC22A5* gene yielded the mutation c.1319C > T in the homozygous state. Both parents were identified as being heterozygous for this gene variant. Patient 2’s echocardiographs during her newborn period were unremarkable. Carnitine supplementation (100 mg/kg/day) in patient 2 was started immediately, and the free carnitine concentration rose to normal values.

**Table 1:** Biochemical parameters at diagnosis and mutation analysis in patients 1 and 2

	Patient 1	Patient 2	Reference range
**Newborn screening**			
Free carnitine in dried blood	1st screening: 3 μmol/l 2nd screening: 2 μmol/l	1st screening: 3 μmol/l 2nd screening: 1 μmol/l	6–65 μmol/l
**Confirmation diagnostics**	**At age 3 weeks**		
Free carnitine in serum	12 μmol/l		12–46 μmol/l
	**At age 7 years**	**At age 1 month**	
Free carnitine in dried blood	1.3 μmol/l	0.93 μmol/l	15–60 μmol/l
Free carnitine in serum	n.a.	3.76 μmol/l	15–68 μmol/l
Free carnitine in urine (mmol/mol creatinine)	39.7 mmol/mol creatinine	22.2 mmol/mol creatinine	< 41.5 mmol/mol creatinine
renal carnitine reabsorption	n.a.	72%	> 98%
*SLC22A5* gene mutation	homozygous for c.1319C > T; p.Thr440Met	homozygous for c.1319C > T; p.Thr440Met	

Following patient 2’s diagnosis, the whole family was screened. While the two oldest children of the family presented normal carnitine concentrations in dried blood spots and urine, patient 1 was also diagnosed with PCD (see Table 1). Genetic analysis revealed the same homozygous gene variant as his sister’s, and he was also put on carnitine supplementation (100 mg/kg/day). Reassessment of patient 1’s newborn screens revealed that free carnitine in his dried blood had been decreased in both the first and requested follow-up screening card (Table 1). Confirmatory testing was recommended, and another biochemical investigation was initiated by a general paediatric hospital. His free carnitine in serum was within the lower limit of the reference range. Carnitine reabsorption in urine was not measured at that time. No molecular or enzymatic investigations were conducted. The child was not referred to a metabolic centre.

## Discussion

PCD is a rare but well-treatable, inborn metabolic disorder caused by mutations in the *SLC22A5* gene. As carnitine plays a crucial role in transporting long-chain fatty acids to the mitochondria, systemic carnitine deficiency leads to impaired long-chain fatty acid oxidation with consecutive energy deficiency. The cardiac muscle obtains 50–70% of its energy from fatty acids, thus the heart is one of the main affected organs in PCD [1]. Cardiomyopathy usually develops between 1 and 4 years of age, as was the case in our patient. OCTN2-associated cardiomyopathy responds poorly to standard therapy, and if the condition is not diagnosed correctly and no carnitine is administered, progressive heart failure may lead to transplantation or death [1].

Cardiomyopathy in PCD can be either dilated or hypertrophic [9]. Histological data are only available on a few patients [10]. Matsuishi et al. report excessive lipid droplets in the cardiac muscle biopsy of a Japanese patient [10]. Cardiomyopathy in PCD has been well-characterised in the JVS mouse model. Kuwajima et al. demonstrated that the total carnitine content in JVS mouse heart was about 10% of that of control mouse heart at 4 and 8 weeks of age [11]. The JVS mice developed hypertrophic cardiomyopathy, a higher cardiac weight/body weight ratio, and larger wall areas of both ventricles and septum in JVS mice. Histologically, the myocyte diameter was increased in JVS mice. Electron microscopy studies revealed a significant increase in mitochondrial mass as well as a six-fold higher lipid fraction compared to control mice. Hypertrophy was associated with lower levels of ATP and ADP, and adenylate energy charge [11]. We were unfortunately unable to conduct any biochemical investigations or electron microscopy in patient 1’s explanted heart as only paraffin samples had been conserved. However, his muscle histology displayed in Fig. 1 showed anomalies that closely resembled those detected in the mouse model.

According to the evidence from children and adults, cardiomyopathy in PCD responds well to carnitine supplementation, and is rapidly reversible under treatment [12, 13]. Wang et al. reported a series of six children with PCD who all presented with severe left ventricular dysfunction. After one month of carnitine treatment, left ventricular systolic function normalised in all six patients, and after six months of therapy, left ventricular volume normalised also [12]. Similar observations have been made in a 24-year-old OCTN2-deficient woman who developed severe dilated left-ventricular cardiomyopathy 3 months after discontinuing carnitine therapy. She demonstrated a dramatic improvement in biventricular function entailing normalised left and right ventricular systolic function just 5 days after re-initiating carnitine supplementation. Carnitine therapy might well have succeeded even in our patient with life-threatening cardiomyopathy, and, had he not required bridging with an assist device to alleviate severe cardiomyopathy and cardiac failure, could have rendered cardiac transplant obsolete. As carnitine supplementation can both prevent clinical symptoms from developing in asymptomatic patients and reverse even severe cardiac symptoms, we believe that PCD should be ruled out immediately in *every* patient presenting cardiomyopathy, and diagnostic gene panels for cardiomyopathy should include the *SLC22A5* gene.

It is difficult to speculate about what would have happened to patient 1’s transplanted heart in the PCD setting over the longterm had carnitine supplementation not been started. The transplanted heart expresses a functional OCTN2 protein, however, the carnitine concentration in serum was still extremely low. The first three enzymes in endogenous carnitine biosynthesis are expressed in all body tissues, while the γ − butyrobetaine hydroxylase that catalyses the last step in carnitine biosynthesis is only present in kidney, liver, and brain tissues [14]. Therefore, the heart and skeletal muscle cannot endogenously synthesise carnitine and thus rely entirely on carnitine import for long chain fatty acid oxidation [1]. There is ample evidence that carnitine depletion can cause intracellular accumulation of fatty acids, decreased detoxification and removal of toxic acyl groups from mitochondria, and an increase in myocardial reactive oxygen species that may be arrhythmogenic [15]. We can thus assume that the transplanted organ would still have been at risk of cardiac arrhythmia.

Untreated patients with PCD carry a potentially fatal risk for cardiac arrhythmias. Both long-QT and short QT syndromes have been observed [1]. De Biase et al. reported a female patient who presented with long QT syndrome leading to a syncopal episode due to ventricular tachycardia in her early twenties [16]. She was diagnosed with PCD after her newborn daughter screened positive for low free carnitine. Under treatment with L-carnitine, no further syncopal episodes occurred and her QT interval returned to normal. The association between PCD and short-QT syndrome was recently reported in a very few patients [17, 18]. The relationship between PCD, a short QT syndrome and arrhythmias was studied by Roussel et al. in a mouse model of carnitine deficiency induced by long-term subcutaneous perfusion of MET88 [17]. They showed that MET88-treated mice developed cardiac hypertrophy associated with a remodelled mitochondrial network. Continuous electrocardiogramme monitoring confirmed a shortened QT interval that correlated negatively with the plasma carnitine concentration, anomalies that coincided with the genesis of ventricular premature beats, ventricular tachycardia, and fibrillation. Data from patients on the Faroe Islands (where PCD has an incidence of about 1:300) reveal that even asymptomatic adult patients are at risk of sudden death from cardiac arrhythmia. In a study by Rasmussen et al., all medico-legal cases of sudden death between 1979 and 2012 among subjects below age 45 years were systematically investigated [15], and the authors demonstrated a strong association between sudden death and untreated PCD, especially in females.

Our patients were homozygous for the variant c.1319C > T in exon 8 of the *SLC22A5* gene. This variant results in a threonine by methionine exchange in position 440 of the OCTN2 protein and was first described by Lamhonwah et al. in 2002 [9]. Frigeni et al. functionally characterised this mutant protein in carnitine-uptake studies, and identified only 0.3% of normal transport activity in fibroblasts in a patient homozygous for this variant [19]. While the c.1319C > T is usually associated with a multisystemic disease, Papadopoulou-Legbelou et al. [20] reported a homozygous patient with a pure cardiac phenotype similar to our patient’s. Their boy presented with dilated cardiomyopathy that was fully reversible under carnitine supplementation [20].

PCD is part of newborn screening programmes in many countries. Patients are identified through significantly reduced free carnitine concentrations of < 2.5–10% of the normal value [3]. The fact that the calculated frequency of mutant alleles based on the frequency of pathogenic alleles as reported in the ExAC Browser Beta and in the gnomAD Browser in about 120,000 healthy (heterozygous) individuals is significantly higher than that reported in newborn screening suggests that the current neonatal screening protocols might be failing to detect some affected individuals [19]. It has been hypothesised that a diagnosis can fail if screening is done too soon after birth and no second screening is obtained, since carnitine is transferred from the mother to her child via the placenta, and the levels of free carnitine shortly after birth tend to reflect maternal carnitine concentrations [21]. Therefore, free-carnitine levels are usually lower in the infants of mothers with PCD immediately after birth than in infants themselves affected by the disease. While the free-carnitine levels decrease in infants with PCD over time, they remain stable or rise slightly in the infants of affected mothers [21]. Data from the Region 4 Stork collaborative project also show that a large proportion of patients with PCD exhibit C0 levels in their first newborn screening sample above the standard cut-offs that apply for low C0 in newborn screening [22]. A study conducted in the Faroe Islands revealed that post-neonatal screening beyond 2 months of age successfully identified additional affected patients whose newborn screening results had been unremarkable [23]. The low sensitivity and specificity of newborn screening for PCD and the numerous asymptomatic mothers identified makes including PCD generally within newborn screening programmes controversial [8]. In our family, patient 2’s having undergone newborn screening for PCD ultimately enabled its diagnosis in two children. Also patient 1, born before the “Newborn Screening 2020” pilot study was initiated, was indeed identified by newborn screening, although PCD is not a target disease incorporated within the German newborn screening programme, since carnitine is essential to interpreting acylcarnitine data with respect to the other fatty acid-oxidation defects included in the newborn screening panel. However, and unexpectedly in a PCD patient, although his carnitine concentration in serum was at the lower limit of the reference range at age 3 weeks, it was not significantly reduced. As carnitine excretion in urine and tubular reabsorption were not measured, this boy was unfortunately not diagnosed until after PCD was confirmed in his baby sister following abnormal newborn-screening findings. As PCD is not included in all newborn screening programmes, and a PCD diagnosis can also be missed during newborn screening, we maintain that OCTN2 deficiency should be ruled out in all patients presenting suggestive symptoms even in the presence of a normal newborn-screening result.

## Conclusion

As OCTN2 deficiency is a readily-treatable disorder with excellent outcome, PCD should be ruled out in every patient revealing cardiomyopathy and cardiac arrhythmias. Family screening is strongly recommended to identify asymptomatic individuals at risk of cardiac manifestations.

## Data Availability

Not applicable.

## Abbreviations

ATB^0,+^: Amino acid transporter B^0,+^
eCPR: Extracorporeal cardiorespiratory resuscitation
HTx: Heart transplantation
OCTN2: Organic cation transporter novel 2
PCD: Primary carnitine deficiency
TMAO: Trimethylamine-N-oxide
